# Synthesis and In Vitro Antimycobacterial and Antibacterial Activity of 8-OMe Ciprofloxacin-Hydrozone/Azole Hybrids

**DOI:** 10.3390/molecules22071171

**Published:** 2017-07-13

**Authors:** Zhi Xu, Shu Zhang, Lian-Shun Feng, Xiao-Ning Li, Guo-Cheng Huang, Yun Chai, Zao-Sheng Lv, Hui-Yuan Guo, Ming-Liang Liu

**Affiliations:** 1 Key Laboratory of Hubei Province for Coal Conversion and New Carbon Materials, Wuhan University of Science and Technology, Wuhan 430081, China; chemorgchem@126.com (Z.X.); drugscience@163.com (G.-C.H.); 2 Institute of Medicinal Biotechnology, Chinese Academy of Medical Sciences and Peking Union Medical College, Beijing 100050, China; ochemistry@163.com (L.-S.F.); whorgchem@126.com (X.-N.L.); chemmedchem@163.com (Y.C.); guohuiyuan108@126.com (H.-Y.G.); 3 Pony Testing International Group (Wuhan), Wuhan 430000, China; zhangshu-zs@163.com

**Keywords:** 8-OMe ciprofloxacin, hybrids, synthesis, antimycobacterial, antibacterial

## Abstract

A series of novel 8-OMe ciprofloxacin (CPFX)-hydrazone/azole hybrids were designed, synthesized, and evaluated for their in vitro biological activities. Our results reveal that all of the hydrozone-containing hybrids (except for **7**) show potency against *Mycobacterium tuberculosis* (MTB) H_37_Rv (minimum inhibitory concentration (MIC): <0.5 μM), which is better than the parent drug CPFX, and comparable to moxifloxacin and isoniazid, some of the tested Gram-positive strains (MIC: 0.06–4 μg/mL), and most Gram-negative strains (MIC: ≤0.03–4 μg/mL).

## 1. Introduction

Fluoroquinolones (FQs) have emerged as a family of synthetic broad spectrum antimicrobial drugs, and the development of FQs was initiated in 1962 with the discovery of nalidixic acid. To date, several generations of FQs have been developed based on their antibacterial spectrum, which is getting significantly broader with each new generation, but there is no standard employed to determine which drug belongs to which generation [1]. The second and the third generations of FQs predominately act on Gram-negative bacteria, some Gram-positive bacteria, and intracellular microbes, while the latest fourth generation FQs are highly active against many species of Gram-positive pathogens, and anaerobic bacteria combined with the above mentioned microbes [2]. Currently, FQs are the second most widely used antimicrobial drugs, with extensive indications for infections including upper and lower respiratory infections, gastrointestinal infections, gynecologic infections, sexually transmitted diseases, prostatitis, and some skin, bone, and soft tissue infections, and their value and role in the treatment of bacterial infections continues to expand [1,3,4].

These antimicrobial drugs act by binding two type II bacterial topoisomerase enzymes, DNA gyrase and topoisomerase IV, thereby inhibiting DNA replication and transcription: for most Gram-negative bacteria, DNA gyrase is the target, whereas topoisomerase IV is the target for many Gram-positive bacteria [5]. It is believed that eukaryotic cells do not contain DNA gyrase or topoisomerase IV, while recent evidence has shown eukaryotic topoisomerase II is also a target for a variety of quinolone-based drugs [6]. The fourth generation FQs act at both DNA gyrase and topoisomerase IV, and this dual action slows the development of resistance [7]. Although they share a common/similar mechanism of action, they differ significantly in their antimicrobial spectrum of activity, their pharmacokinetic characteristics, and, to some extent, their safety profiles.

Furthermore, FQs demonstrate potential anti-tuberculosis (TB) activity. Although they are presently used to treat primarily in cases involving the resistance or intolerance to first-line anti-TB therapy by the World Heath Organization (WHO) [8], these drugs are potential first-line agents and are under study for this indication [9]. With increasing numbers of FQ prescriptions and the expanded use of these broad-spectrum agents, the selective pressure of FQ use results in the ready emergence of FQ resistance in a diversity of organisms, including *Mycobacterium tuberculosis* (MTB) and multidrug-resistant gram-positive bacteria, such as the methicillin-resistant *Staphylococcus aureus* (MRSA). For MTB, resistance is emerging and may herald a significant future threat to the long-term clinical utility of FQs [9]. All of the above facts necessitate an urgent need to develop new agents with a unique mechanism of action different from that of the currently used anti-bacterial/anti-TB drugs, and which are fast acting, well-tolerated, effective against both drug-susceptible and drug-resistant strains of organisms, of low toxicity, and of short therapy duration.

On the other hand, the extreme complexity and hydrophobicity of the cell envelop of mycobacteria is a barrier that prevents many agents from penetrating into the bacteria, and thus unable to access the intended targets. To some extent, the lipophilicity of FQs plays an important role in their penetration into mycobacterial cells, and simply increasing the lipophilic character may also increase the anti-TB activity [10,11,12]. Therefore, the strategy to increase the lipophilicity of FQs may lead to promising anti-TB candidates.

**Figure 1:**
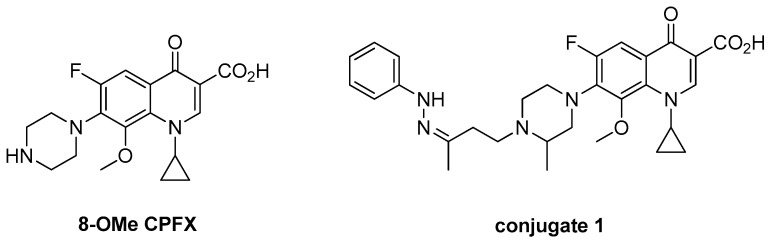
Chemical structures of 8-OMe CPFX and conjugate 1.

The structure-activity relationship (SAR) reveals that the introduction of the OMe group at the C-8 position of the FQ motif has resulted in a greater binding affinity to the topoisomerase IV enzyme, which results in enhanced activity against gram-positive pathogens and anaerobes while maintaining excellent potency against gram-negative organisms, as evidenced by gatifloxacin (GTFX) and moxifloxacin (MXFX). Interestingly, 8-OMe FQ derivatives with an N1-cyclopropyl substitution are much more potent against resistant MTB than their 8-H analogs [13]. It is noted that some of the 8-OMe ciprofloxacin (8-OMe CPFX, Figure 1) derivatives exhibit considerable antibacterial/anti-TB activity [14,15], which warrants further investigation.

Azoles, especially imidazole and triazole, are very useful pharmacophores due to their various biological activities, and some of them are currently used in the clinic for the treatment of common diseases [16,17]. It is notable that CPFX-azole hybrids exhibit excellent activity against both drug-susceptible and drug-resistant bacteria, and some of them are far more potent than the parent CPFX [18,19,20,21,22,23]. Moreover, our previous study demonstrated that an incorporation of hydrozone motif into GTFX could lead to an improvement of the activity against both Gram-positive and Gram-negative bacteria, and conjugate 1 (Figure 1), as the most emblematic example, has a broad antimicrobial spectrum with a minimum inhibitory concentration (MIC) in a range of 0.06–1 μg/mL [24].

Based on the above research results, and as a part of an ongoing program to optimize 8-OMe FQ derivatives as anti-bacterial/anti-TB agents [24,25,26,27,28], a series of 8-OMe CPFX-hydrozone/azole hybrids were designed, synthesized, and evaluated for their biological activity in this study. Our primary objective was to optimize the potency of these compounds against clinically important pathogens and MTB. A preliminary SAR study is also explored to facilitate the further development of FQs.

## 2. Results and Discussion

### 2.1. Chemistry

**Scheme 1:**
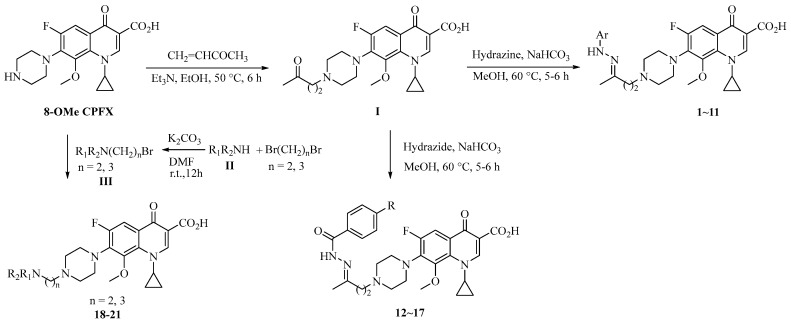
Synthesis of 8-OMe CPFX-containing hybrids **1**–**21**.

Detailed synthetic pathways for the desired targets **1**–**21** are outlined in Scheme 1. The alkylation of 8-OMe CPFX with but-3-en-2-one gave intermediate **I** (yield: 50%). Subsequently, the treatment of the ketone **I** with various hydrazine hydrochlorides in the presence of NaHCO_3_ or free hydrazines or hydrazides in methanol at 60 °C provided the hydrozone hybrids **1**–**11** and acylhydrazones **12**–**17** (yields: 29–61%) [24].

For the four azole-containing hybrids **18**–**21**, azole **II** was first alkylated with 1,2-dibromoethane or 1,3-dibromopropane in the presence of K_2_CO_3_ to afford the corresponding *N*-(2-bromoethyl/3-bromopropyl)azole **III** (yields: 42–65%). The desired hybrids **18**–**21** (yields: 44–51%) were obtained by the treatment of **III** with 8-OMe CPFX at room temperature in the presence of K_2_CO_3_ [15].

### 2.2. Anti-MTB Activity

The synthesized hybrids were preliminarily screened for in vitro activity against the MTB H_37_Rv ATCC27294 strain, using the Microplate Alamar Blue Assay (MABA) [29]. The minimum inhibitory concentration is defined as the lowest concentration effecting a reduction in fluorescence of >90% relative to the mean of replicate bacterium-only controls. The MIC values of the compounds along with CPFX, MXFX, and isoniazid (INH) for comparison are presented in µM in Table 1.

The data reveals that the activity of the azole-containing hybrids **18**–**21** is generally poor (MIC: >1.7 μM) against this strain, but all of the hydrozone-containing hybrids with the exception only of (**7**) display considerable activity (MIC: <0.5 μM), which is more than the parent drug CPFX (MIC: 1.30 μM). Among them, compounds **3** and **11**–**16** have better activity (MIC: 0.210–0.272 μM) than MXFX (MIC: 0.289 μM), the most active anti-TB FQ, and INH (MIC: 0.336 μM). The potency of the hydrozones **1**–**11** is related to the aromatic moiety (Ar). For example, when the F atom on the benzene ring of the most active hydrozone, **3** (MIC: 0.215 μM), is replaced with an electron-donating Me (**1**) or OMe (**2**), or other electron-withdrawing Cl (**4**), CF_3_ (**5**), or 3-Cl-4-F (**6**), it results in slightly decreased activity. Interestingly, 2-pyridyl derivative is much more active than the corresponding 4-pyridyl one (**7** vs. **8**). Moreover, the relative order of the aromatic heterocycles affecting activity is 7-chloroquinoline > benzo[*d*]thiazole > 1*H*-benzo[*d*]imidazole (**9** vs. **10** vs. **11**). For acylhydrazones **12**–**17**, the introduction of an electron-donating group on the benzene ring improves the activity (**12** vs. **13**, **14**), while an electron-withdrawing one is detrimental to the potency (**12** vs. **15**–**17**). The azole-containing hydrids **18**–**21** show much less activity than CPFX, and the activity of the linkers between CPFX and azole was ethylene >> propylidyne (**18** vs. **19**).

### 2.3. Antibacterial Activity

The target hybrids **1**–**21** were evaluated for their in vitro antibacterial activity against representative strains using standard techniques [24]. The minimum inhibitory concentration (MIC) is obtained from three independent experiments, defined as the concentration of the compound required to give complete inhibition of bacterial growth, and the MIC values of **1**–**21** against Gram-positive and Gram-negative strains, along with those of CPFX and levofloxacin (LVFX) for comparison, are listed in Table 2 and Table 3, respectively. These data indicate that all of the target hybrids except for **7** and **19**–**21** have a similar antibacterial spectrum to CPFX and LVFX. These hybrids exhibit considerable potency in inhibiting the growth of some tested Gram-positive strains, such as the methicillin-sensitive *Staphylococcus epidermidis* (MSSE), the methicillin-sensitive *S. aureus* (MSSA), MRSA, and *Enterococcus faecalis* (two strains) (MIC: 0.06–4 μg/mL), as well as most of the tested Gram-negative strains (MIC: ≤0.03–4 μg/mL). It is worth noting that compound **16** shows useful activity (MIC: 0.5 μg/mL) against the CPFX-resistant *Stenotrophomonas maltophilia*, a common clinical pathogen.

Generally, hybrids **1**–**21** share a similar antibacterial trend with that of anti-MTB, i.e., the activity order of the (hetero)aromatic rings against both Gram-positive and -negative strains was in the order: acylhydrazones ≥ hydrazones >> azoles. In addition, ethylene imidazole hybrid **18** is much more potent than the corresponding propylidyne imidazole analog **19** and the ethylene triazole hybrids **20** and **21**.

## 3. Experimental Section

### 3.1. General

Melting points were determined in open capillaries and uncorrected. Clog P was calculated by CLOGP module in sybyl 7.3 software. ^1^H-NMR spectra were determined on a Varian Mercury-400 spectrometer (Varian Medical Systems Inc., Palo Alto, CA, USA)in DMSO-*d*_6_, CD_3_OD, or CDCl_3_ using tetra-methylsilane (TMS) as an internal standard. Electro spray ionization (ESI) mass spectra were obtained on a MDSSCIEXQ-Tap mass spectrometer (Thermo Fisher Scientific, San Jose, CA, USA). Unless otherwise noted, the reagents were obtained from a commercial supplier and were used without further purification. 

### 3.2. Synthesis

#### 3.2.1. Method 1

A mixture of 8-OMe CPFX (10.0 mmol), but-3-en-2-one (20.0 mmol), and triethylamine (20.0 mmol) in anhydrous ethanol (25 mL) was stirred for 6 h at 50 °C under an atmosphere of nitrogen. The precipitate obtained was filtered and recrystallized from methanol to give 1-cyclopropyl-6-fluoro-8-methoxy-4-oxo-7-(4-(3-oxobutyl)piperazin-1-yl)-1,4-dihydroquinoline-3-carboxylic acid **I** as an off-white solid. Yield: 50%. ^1^H-NMR (500 MHz, CDCl_3_) δ 0.99–0.98 (2H, m, cyclopropyl-H, CH_2_), 1.21–1.20 (2H, m, cyclopropyl-H, CH_2_), 2.21 (3H, s, CH_3_), 2.79–2.67 (8H, m), 3.46 (4H, brs), 3.78 (3H, s, OCH_3_), 4.01–4.02 (1H, m, cyclopropyl-H), 7.83 (1H, d, Ar-H), 8.79 (1H, s, Ar-H), 14.78 (1H, brs, COOH). MS-ESI (*m*/*z*): 432.5 (M + H)^+^.

To a solution of **I** (0.11 mmol) in methanol (2 mL) was added a mixture of hydrazine hydrochloride (0.11 mmol) and NaHCO_3_ (0.12 mmol) in H_2_O (1 mL) at room temperature. The reaction mixture was heated to 60 °C and stirred for 5–6 h and concentrated under reduced pressure. The precipitate was filtered and recrystallized from methanol (1 mL) to give targets **1**–**6** (yield: 45–61%) as off-white solids.

#### 3.2.2. Method 2 

A mixture of **I** (0.11 mmol) and hydrazide or hydrazine (0.11 mmol) in anhydrous methanol (2 mL) was stirred for 5–6 h at 60 °C under an N_2_ atmosphere. The precipitate was filtered and recrystallized from methanol (1 mL) to give targets **7**–**17** (yield: 29–57%) as off-white solids.

#### 3.2.3. Method 3

A mixture of azole **II** (1 mmol), 1,2-dibromoethane (5 mmol) or 1,3-dibromopropane (5 mmol), and K_2_CO_3_ (10 mmol) in DMF (20 mL) was stirred at room temperature overnight. After filtration, the mixture was diluted with dichloromethane (DCM, 100 mL) and washed with H_2_O (100 mL × 3). After the removal of the solvent, crude *N*-(2-bromoethyl/3-bromopropyl)azole **III** (yield: 42–65%) was obtained as a colorless oil, which was used directly in the next step. A mixture of **III** (0.2 mmol), 8-OMe CPFX (0.2 mmol), and K_2_CO_3_ (1 mmol) in DMF (5 mL) was stirred at room temperature overnight. After filtration, the mixture was concentrated under reduced pressure. The residue was purified by silica gel chromatography eluted with DCM to DCM:MeOH = 10:1 to give targets **18**–**21** (yield: 44–51%) as off-white solids.

*1-Cyclopropyl-6-fluoro-8-methoxy-4-oxo-7-(4-(3-(2-(p-tolyl)hydrazono)butyl)-piperazin-1-yl)-1,4-dihydroquinoline-3-carboxylic acid* (**1**). 47% yield, method 1. M.p.: 180–184 °C. ^1^H-NMR (500 MHz, CD_3_OD) δ_H_ 1.08–1.09 (2H, m, cyclopropyl-H, CH_2_), 1.25–1.27 (2H, m, cyclopropyl-H, CH_2_), 1.99 (3H, s, CH_3_), 2.27 (3H, s, CH_3_), 2.61–2.64 (2H, m, CH_2_), 2.72–2.87 (6H, m, 3CH_2_), 3.51–3.54 (4H, m, 2CH_2_), 3.87 (3H, s, OCH_3_), 4.24 (1H, s, cyclopropyl-H), 7.01–7.03 (4H, m, Ar-H), 7.85–7.88 (1H, m, Ar-H), 8.90 (1H, s, Ar-H). ESI-MS: *m*/*z* 536.3 [M + H]^+^.

*1-Cyclopropyl-6-fluoro-8-methoxy-7-(4-(3-(2-(3-methoxyphenyl)hydrazono)-butyl)piperazin-1-yl)-4-oxo-1,4-dihydroquinoline-3-carboxylic acid* (**2**). 49% yield, method 1. M.p.: 177–179 °C. ^1^H-NMR (500 MHz, CD_3_OD) δ_H_ 1.07–1.08 (2H, m, cyclopropyl-H, CH_2_), 1.24–1.25 (2H, m, cyclopropyl-H, CH_2_), 1.99 (3H, s, CH_3_), 2.61–2.85 (8H, m, 4CH_2_), 3.50–3.53 (4H, m, 2CH_2_), 3.80 (3H, s, OCH_3_), 3.86 (3H, s, OCH_3_), 4.22 (1H, m, cyclopropyl-H), 6.35–6.36 (1H, m, Ar-H), 6.66–6.67 (1H, m, Ar-H), 6.74 (1H, s, Ar-H), 7.07–7.10 (1H, m, Ar-H), 7.84–7.87 (1H, m, Ar-H), 8.85 (1H, s, Ar-H). ESI-MS: *m*/*z* 552.3 [M + H]^+^.

*1-Cyclopropyl-6-fluoro-7-(4-(3-(2-(4-fluorophenyl)hydrazono)butyl)piperazin-1-yl)-8-methoxy-4-oxo-1,4-dihydroquinoline-3-carboxylic acid* (**3**). 50% yield, method 1. M.p.: 186–188 °C. ^1^H-NMR (500 MHz, CD_3_OD) δ_H_ 1.09 (2 H, s, cyclopropyl-H, CH_2_), 1.26–1.27 (2H, m, cyclopropyl-H, CH_2_), 2.00–2.09 (3H, m, CH_3_), 2.62–2.87 (8H, m, 4CH_2_), 3.54 (4H, s, 2CH_2_), 3.87 (3H, s, OCH_3_), 4.25 (1H, s, cyclopropyl-H), 6.93–6.96 (2H, m, Ar-H), 7.07–7.08 (2H, m, Ar-H), 7.86–7.88 (1H, m, Ar-H), 8.90 (1H, s, Ar-H). ^13^C-NMR (100 MHz, DMSO-*d*_6_) δ 9.44, 16.19, 36.22, 41.28, 50.76, 53.70, 55.75, 63.16, 107.06, 113.65, 115.58, 121.22, 134.58, 139.62, 143.93, 145.70, 146.26, 150.98, 154.76, 157.08, 157.22, 166.14, 176.77. ESI-MS: *m*/*z* 540.3 [M + H]^+^.

*7-(4-(3-(2-(4-Chlorophenyl)hydrazono)butyl)piperazin-1-yl)-1-cyclopropyl-6-fluoro-8-methoxy-4-oxo-1,4-dihydroquinoline-3-carboxylic acid* (**4**). 61% yield, method 1. M.p.: 189–191 °C. ^1^H-NMR (500 MHz, CD_3_OD) δ_H_ 1.09 (2H, s, cyclopropyl-H, CH_2_), 1.26–1.27 (2H, m, cyclopropyl-H, CH_2_), 2.00 (3H, s, CH_3_), 2.72–2.87 (8H, m, 4CH_2_), 3.55 (4H, m, 2CH_2_), 3.87 (3H, s, OCH_3_), 4.25 (1H, s, cyclopropyl-H), 7.07–7.09 (2H, m, Ar-H), 7.16–7.18 (2H, m, Ar-H), 7.86–7.88 (1H, m, Ar-H), 8.90 (1H, s, Ar-H). ESI-MS: *m*/*z* 556.3 [M + H]^+^.

*1-Cyclopropyl-6-fluoro-8-methoxy-4-oxo-7-(4-(3-(2-(4-(trifluoromethyl)phenyl)hydrazono)butyl)piperazin-1-yl)-1,4-dihydroquinoline-3-carboxylic acid* (**5**). 48% yield, method 1. M.p.: 198–200 °C. ^1^H-NMR (500 MHz, DMSO-*d*_6_) δ_H_ 1.05 (2H, s, cyclopropyl-H, CH_2_), 1.14–1.15 (2H, m, cyclopropyl-H, CH_2_), 1.97–2.04 (3H, m, CH_3_), 2.64–2.68 (8H, m, 4CH_2_), 3.32–3.37 (4H, m, 2CH_2_), 3.83 (3H, s, OCH_3_), 4.19 (1H, s, cyclopropyl-H),7.20–7.21 (2H, m, Ar-H), 7.49–7.51 (2H, m, Ar-H), 7.76–7.78 (1H, m, Ar-H), 8.72 (1H, s, Ar-H), 9.26 (1H, s, NH). ESI-MS: *m*/*z* 590.4 [M + H]^+^.

*7-(4-(3-(2-(3-Chloro-4-fluorophenyl)hydrazono)butyl)piperazin-1-yl)-1-cyclopropyl-6-fluoro-8-methoxy-4-oxo-1,4-dihydroquinoline-3-carboxylic acid* (**6**). 51% yield, method 1. M.p.: 218–220 °C. ^1^H-NMR (500 MHz, CD_3_OD) δ_H_ 1.08–1.09 (2H, m, cyclopropyl-H, CH_2_), 1.25–1.26 (2H, m, cyclopropyl-H, CH_2_), 1.99–2.09 (3H, m, CH_3_), 2.63–2.86 (8H, m, 4CH_2_), 3.51–3.54 (4H, m, 2CH_2_), 3.87 (3H, s, OCH_3_), 4.24 (1H, s, cyclopropyl-H), 6.99 (1H, s, Ar-H), 7.04–7.08 (1H, m, Ar-H), 7.19–7.20 (1H, m, Ar-H), 7.85–7.88 (1H, m, Ar-H), 8.90 (1H, s, Ar-H). ESI-MS: *m*/*z* 574.3 [M + H]^+^.

*1-Cyclopropyl-6-fluoro-8-methoxy-4-oxo-7-(4-(3-(2-(pyridin-4-yl)hydrazono)-butyl)piperazin-1-yl)-1,4-dihydroquinoline-3-carboxylic acid* (**7**). 40% yield, method 2. M.p.: 299–300 °C. ^1^H-NMR (500 MHz, CD_3_OD) δ_H_ 1.08–1.09 (2H, m, cyclopropyl-H, CH_2_), 1.25–1.27 (2H, m, cyclopropyl-H, CH_2_), 2.10–2.13 (3H, m, CH_3_), 2.69–2.71 (2H, m, CH_2_), 2.80–2.81 (4H, m, 2CH_2_), 2.86–2.89 (2H, m, CH_2_), 3.49–3.53 (4H, m, 2CH_2_), 3.87 (3H, s, OCH_3_), 4.24 (1H, s, cyclopropyl-H), 7.23 (2H, s, Ar-H), 7.86–7.88 (1H, m, Ar-H), 8.17–8.18 (2H, m, Ar-H), 8.90 (1H, s, Ar-H). ESI-MS: *m*/*z* 523.5 [M + H]^+^.

*1-Cyclopropyl-6-fluoro-8-methoxy-4-oxo-7-(4-(3-(2-(pyridin-2-yl)hydrazono)-butyl)piperazin-1-yl)-1,4-dihydroquinoline-3-carboxylic acid* (**8**). 32% yield, method 2. M.p.: 142–145 °C. ^1^H-NMR (500 MHz, CD_3_OD) δ_H_ 1.05–1.06 (2H, m, cyclopropyl-H, CH_2_), 1.21–1.22 (2H, m, cyclopropyl-H, CH_2_), 2.04–2.05 (3H, m, CH_3_), 2.66–2.67 (2H, m, CH_2_), 2.76–2.86 (6H, m, 3CH_2_), 3.51–3.56 (4H, m, 2CH_2_), 3.83 (3H, s, OCH_3_), 4.12–4.16 (1H, m, cyclopropyl-H), 6.77–6.80 (1H, m, Ar-H), 7.18–7.23 (1H, m, Ar-H), 7.62–7.67 (1H, m, Ar-H), 7.83–7.85 (1H, m, Ar-H), 8.07 (1H, s, Ar-H), 8.75 (1H, s, Ar-H). ESI-MS: *m*/*z* 523.5 [M + H]^+^.

*7-(4-(3-(2-(1H-Benzo[d]imidazol-2-yl)hydrazono)butyl)piperazin-1-yl)-1-cyclopropyl-6-fluoro-8-methoxy-4-oxo-1,4-dihydroquinoline-3-carboxylic acid* (**9**). 29% yield, method 2. M.p.: 249–251 °C. ^1^H-NMR (500 MHz, CDCl_3_) δ_H_ 1.01–1.02 (2H, m, cyclopropyl-H, CH_2_), 1.10–1.11 (2H, m, cyclopropyl-H, CH_2_), 2.06 (3H, s, CH_3_), 2.50–2.70 (8H, m, 4CH_2_), 3.48–3.53 (4H, m, 2CH_2_), 3.83 (3H, s, OCH_3_), 4.17 (1H, s, cyclopropyl-H), 6.87–6.92 (2H, m, Ar-H), 7.09–7.19 (2H, m, Ar-H), 7.74–7.76 (1H, m, Ar-H), 8.69 (1H, s, Ar-H), 11.23 (1H, brs, COOH). ESI-MS: *m*/*z* 562.5 [M + H]^+^.

*7-(4-(3-(2-(Benzo[d]thiazol-2-yl)hydrazono)butyl)piperazin-1-yl)-1-cyclopropyl-6-fluoro-8-methoxy-4-oxo-1,4-dihydroquinoline-3-carboxylic acid* (**10**). 57% yield, method 2. M.p.: 212–214 °C. ^1^H-NMR (500 MHz, CD_3_OD) δ_H_ 1.09–1.10 (2H, m, cyclopropyl-H, CH_2_), 1.25–1.27 (2H, m, cyclopropyl-H, CH_2_), 2.18 (3H, s, CH_3_), 2.73–2.90 (8H, m, 4CH_2_), 3.60–3.66 (4H, m, 2CH_2_), 3.90 (3H, s, OCH_3_), 4.26 (1H, s, cyclopropyl-H), 7.13–7.40 (4H, m, Ar-H), 7.87–7.92 (1H, m, Ar-H), 8.91 (1H, s, Ar-H). ESI-MS: *m*/*z* 579.22 [M + H]^+^.

*7-(4-(3-(2-(7-Chloroquinolin-4-yl)hydrazono)butyl)piperazin-1-yl)-1-cyclopropyl-6-fluoro-8-methoxy-4-oxo-1,4-dihydroquinoline-3-carboxylic acid* (**11**). 49% yield, method 2. M.p.: 220–222 °C. ^1^H-NMR (500 MHz, CD_3_OD) δ_H_ 1.08–1.09 (2H, m, cyclopropyl-H, CH_2_), 1.25–1.27 (2H, m, cyclopropyl-H, CH_2_), 2.22 (3H, s, CH_3_), 2.76–2.94 (8H, m, 4CH_2_), 3.55 (4H, s, 2CH_2_), 3.87 (3H, s, OCH_3_), 4.24 (1H, s, cyclopropyl-H),7.48–7.50 (2H, m, Ar-H), 7.85–7.87 (2H, m, Ar-H), 8.26–8.27 (1H, m, Ar-H), 8.89 (1H, s, Ar-H). ^13^C-NMR (100 MHz, DMSO-*d*_6_) δ 9.44, 17.02, 36.48, 41.29, 50.77, 53.70, 55.32, 63.18, 102.20, 107.10, 116.47, 121.22, 124.83, 125.12, 127.94, 134.05, 134.59, 139.62, 146.26, 148.61, 149.64, 151.00, 152.42, 154.76, 155.60, 157.24, 166.14, 176.76. ESI-MS: *m*/*z* 607.4 [M + H]^+^.

*7-(4-(3-(2-Benzoylhydrazono)butyl)piperazin-1-yl)-1-cyclopropyl-6-fluoro-8-methoxy-4-oxo-1,4-dihydroquinoline-3-carboxylic acid* (**12**). 35% yield, method 2. M.p.: 152–154 °C. ^1^H-NMR (500 MHz, DMSO-*d*_6_) δ_H_ 1.04–1.07 (2H, m, cyclopropyl-H, CH_2_), 1.12–1.14 (2H, m, cyclopropyl-H, CH_2_), 2.01–2.07 (3H, m, CH_3_), 2.57–2.68 (6H, m, 3CH_2_), 3.25–3.26 (4H, m, 2CH_2_), 3.61–3.64 (2H, m, 2CH_2_), 3.76–3.82 (2H, m, CH_2_), 3.86 (3H, s, OCH_3_), 4.17 (1H, m, cyclopropyl-H), 7.47–7.54 (3H, m, Ar-H), 7.74–7.85 (3H, m, Ar-H), 8.69–8.73 (1H, m, Ar-H), 10.51 (1H, brs, COOH). ESI-MS: *m*/*z* 550.4 [M + H]^+^ .

*1-Cyclopropyl-6-fluoro-8-methoxy-7-(4-(3-(2-(4-methoxybenzoyl)hydrazono)-butyl)piperazin-1-yl)-4-oxo-1,4-dihydroquinoline-3-carboxylic acid* (**13**). 29% yield, method 2. M.p.: 178–180 °C. ^1^H-NMR (500 MHz, CD_3_OD) δ_H_ 1.06–1.08 (2H, m, cyclopropyl-H, CH_2_), 1.22–1.26 (2H, m, cyclopropyl-H, CH_2_), 2.15–2.25 (3H, m, CH_3_), 2.76–2.87 (8H, m, 4CH_2_), 3.54–3.68 (4H, m, 2CH_2_), 3.88–3.91 (6H, m, 2OCH_3_), 4.24 (1H, s, cyclopropyl-H), 7.06–7.10 (2H, m, Ar-H), 7.81–7.93 (3H, m, Ar-H), 8.86–8.88 (1H, m, Ar-H). ESI-MS: *m*/*z* 580.5 [M + H]^+^.

*1-Cyclopropyl-6-fluoro-7-(4-(3-(2-(4-hydroxybenzoyl)hydrazono)butyl)-piperazin-1-yl)-8-methoxy-4-oxo-1,4-dihydroquinoline-3-carboxylic acid* (**14**). 36% yield, method 2. M.p.: 171–173 °C. ^1^H-NMR (500 MHz, CD_3_OD) δ_H_ 1.05–1.08 (2H, m, cyclopropyl-H, CH_2_), 1.22–1.26 (2H, m, cyclopropyl-H, CH_2_), 2.14 (3H, s, CH_3_), 2.72–2.88 (8H, m, 4CH_2_), 3.52–3.54 (4H, m, 2CH_2_), 3.89 (3H, s, OCH_3_), 4.24 (1H, s, cyclopropyl-H), 6.85–6.93 (2H, m, Ar-H), 7.80–7.89 (3H, m, Ar-H), 8.86–8.89 (1H, m). ^13^C-NMR (100 MHz, DMSO-*d*_6_) δ 9.44, 17.21, 36.43, 41.28, 50.75, 53.62, 55.38, 63.19, 106.98, 107.16, 115.23, 121.16, 134.58, 139.54, 139.66, 146.22, 146.27, 150.96, 154.75, 157.23, 160.73, 166.14, 176.75. ESI-MS: *m*/*z* 566.4 [M + H]^+^.

*1-Cyclopropyl-6-fluoro-7-(4-(3-(2-(4-fluorobenzoyl)hydrazono)butyl)piperazin-1-yl)-8-methoxy-4-oxo-1,4-dihydroquinoline-3-carboxylic acid* (**15**). 38% yield, method 2. M.p.: 192–194 °C. ^1^H-NMR (500 MHz, CDCl_3_) δ_H_ 1.02–1.03 (2H, m, cyclopropyl-H, CH_2_), 1.11–1.13 (2H, m, cyclopropyl-H, CH_2_), 2.00 (3H, s, CH_3_), 2.61–2.71 (8H, m, 4CH_2_), 3.31–3.36 (4H, m, 2CH_2_), 3.77 (3H, s, OCH_3_), 4.17 (1H, s, cyclopropyl-H), 7.31 (2H, s, Ar-H), 7.74–7.79 (1H, m, Ar-H), 7.91 (2H, s, Ar-H), 8.70–8.73 (1H, m, Ar-H), 10.48 (1H, brs, COOH). ESI-MS: *m*/*z* 568.4 [M + H]^+^.

*7-(4-(3-(2-(4-Chlorobenzoyl)hydrazono)butyl)piperazin-1-yl)-1-cyclopropyl-6-fluoro-8-methoxy-4-oxo-1,4-dihydroquinoline-3-carboxylic acid* (**16**). 55% yield, method 2. M.p.: 201–203 °C. ^1^H-NMR (500 MHz, DMSO-*d*_6_) δ_H_ 1.05–1.06 (2H, m, cyclopropyl-H, CH_2_), 1.15–1.16 (2H, m, cyclopropyl-H, CH_2_), 2.03 (3H, s, CH_3_), 2.64–2.72 (8H, m, 4CH_2_), 3.39–3.40 (4H, m, 2CH_2_), 3.80 (3H, s, OCH_3_), 4.20 (1H, s, cyclopropyl-H), 7.59 (2H, s, Ar-H), 7.75–7.88 (3H, m, Ar-H), 8.73–8.74 (1H, m, Ar-H), 10.57 (1H, brs, COOH). ESI-MS: *m*/*z* 584.4 [M + H]^+^.

*1-Cyclopropyl-6-fluoro-8-methoxy-7-(4-(3-(2-(4-nitrobenzoyl)hydrazono)-butyl)piperazin-1-yl)-4-oxo-1,4-dihydroquinoline-3-carboxylic acid* (**17**). 30% yield, method 2. M.p.: 187–189 °C. ^1^H-NMR (500 MHz, CD_3_OD) δ_H_ 1.11–1.12 (2H, m, cyclopropyl-H, CH_2_), 1.27–1.28 (2H, m, cyclopropyl-H, CH_2_), 2.07–2.26 (3H, m, CH_3_), 2.67–2.79 (1H, m, CH_2_), 2.94–2.95 (2H, m, CH_2_), 3.45–3.46 (5H, m), 3.76–3.79 (4H, m, 2CH_2_), 3.95 (3H, s, OCH_3_), 4.26 (1H, s, cyclopropyl-H), 7.85–7.92 (1H, m, Ar-H), 8.15–8.16 (2H, m, Ar-H), 8.40–8.42 (2H, m, Ar-H), 8.87–8.92 (1H, m, Ar-H). ESI-MS: *m*/*z* 595.4 [M + H]^+^.

*1-Cyclopropyl-6-fluoro-8-methoxy-7-(4-(2-(2-methyl-5-nitro-1H-imidazol-1-yl)ethyl)piperazin-1-yl)-4-oxo-1,4-dihydroquinoline-3-carboxylic acid* (**18**). 51% yield, method 3. M.p.: 182–189 °C. ^1^H-NMR (500 MHz, CD_3_OD) δ_H_ 1.08–1.14 (2H, m, cyclopropyl-H, CH_2_), 1.28–1.35 (2H, m, cyclopropyl-H, CH_2_), 2.66 (3H, s, CH_3_), 3.45 (4H, s, 2CH_2_), 3.74 (4H, s, 2CH_2_), 3.92–3.94 (4H, m, 2CH_2_), 4.26–4.32 (1H, m, cyclopropyl-H), 4.70 (3H, s, OCH_3_), 7.93–7.98 (1H, m, Ar-H), 8.61 (1H, s, Ar-H), 8.88–9.01 (1H, m, Ar-H). ESI-MS: *m*/*z* 515.4 [M + H]^+^.

*1-Cyclopropyl-6-fluoro-8-methoxy-7-(4-(3-(2-methyl-4-nitro-1H-imidazol-1-yl)propyl)piperazin-1-yl)-4-oxo-1,4-dihydroquinoline-3-carboxylic acid* (**19**). 45% yield, method 3. M.p.: 148–150 °C. ^1^H-NMR (500 MHz, DMSO-*d*_6_) δ_H_ 0.96–0.97 (2H, m, cyclopropyl-H, CH_2_), 1.09–1.10 (2H, m, cyclopropyl-H, CH_2_), 2.17–2.20 (2H, m, CH_2_), 2.43 (3H, s, CH_3_), 2.92 (2H, s, CH_2_), 3.27 (4H, s, 2CH_2_), 3.82 (3H, s, OCH_3_), 4.06 (3H, s), 4.19–4.25 (4H, m, 2CH_2_), 7.65–7.67 (1H, m, Ar-H), 8.43 (2H, s, Ar-H and Imi-H). ESI-MS: *m*/*z* 529.5 [M + H]^+^.

*7-(4-(2-(1H-1,2,4-Triazol-1-yl)ethyl)piperazin-1-yl)-1-cyclopropyl-6-fluoro-8-methoxy-4-oxo-1,4-dihydroquinoline-3-carboxylic acid* (**20**). 44% yield, method 3. M.p.: 158–160 °C. ^1^H-NMR (500 MHz, DMSO-*d*_6_) δ_H_ 0.94–0.98 (2H, m, cyclopropyl-H, CH_2_), 1.07–1.12 (2H, m, cyclopropyl-H, CH_2_), 3.27 (4H, s, 2CH_2_), 3.46–3.52 (4H, m, 2CH_2_), 3.83 (3H, s, OCH_3_), 4.04 (1H, s, cyclopropyl-H), 4.56–4.61 (2H, m, CH_2_), 4.80–4.82 (2H, m, CH_2_), 7.65–7.70 (1H, m, Ar-H), 7.88 (1H, s, triazole), 8.40 (1H, s, Ar-H), 9.28 (1H, s, triazole-H). ESI-MS: *m*/*z* 457.4 [M + H]^+^.

*7-(4-(2-(1H-1,2,3-Triazol-1-yl)ethyl)piperazin-1-yl)-1-cyclopropyl-6-fluoro-8-methoxy-4-oxo-1,4-dihydroquinoline-3-carboxylic acid* (**21**). 50% yield, method 3. M.p.: 280–282 °C. ^1^H-NMR (500 MHz, DMSO-*d*_6_) δ_H_ 0.96–0.97 (2H, m, cyclopropyl-H, CH_2_), 1.11–1.12 (2H, m, cyclopropyl-H, CH_2_), 3.28–3.29 (4H, m, 2CH_2_), 3.48–3.53 (4H, m, 2CH_2_), 3.84 (3H, s, OCH_3_), 4.05 (1H, s, cyclopropyl-H), 4.52–4.53 (2H, m, CH_2_), 4.60–4.61 (2H, m, CH_2_), 7.70–7.72 (1H, m, Ar-H), 8.12–8.17 (1H, m, triazole), 8.48 (1H, s, Ar-H), 8.89 (1H, s, triazole-H). ESI-MS: *m*/*z* 457.7 [M + H]^+^.

### 3.3. Anti-MTB Activity

MICs against replicating *M. tuberculosis* were determined by the microplate Alamar blue assay (MABA) [29]. CPFX, MXFX, and INH were included as positive controls. The range of the final testing concentrations of the targets was 64 to 0.125 μg/mL. *M. tuberculosis* H37Rv was grown to late log phase (70 to 100 Klett units) in Difco Middlebrook 7H9 Broth supplemented with 0.2% (*v*/*v*) glycerol, 0.05% Tween 80, and 10% (*v*/*v*) albumin-dextrosecatalase (BBL Middlebrook ADC Enrichment, catalog No. 212352) (7H9-ADCTG). The cultures were centrifuged, washed twice, and then re-suspended in phosphate buffered saline. The suspensions were then passed through an 8 μM-pore-size filter to remove clumps, and aliquots were frozen at −80 °C. Twofold dilutions of the targets were prepared in 7H9-ADC-TG in a volume of 100 μL in 96-well, black, clear-bottom microplates (BD Biosciences, Franklin Lakes, NJ, USA). *M. tuberculosis* (100 μL containing 2 × 10^5^ CFU) was added, yielding a final testing volume of 200 μL. The plates were incubated at 37 °C; on day seven of incubation, 12.5 μL of 20% Tween 80 and 20 μL of Alamar blue were added to all of the wells. After incubation at 37 °C for 16 to 24 h, the fluorescence was read at an excitation of 530 nm and an emission of 590 nm. The MIC was defined as the lowest concentration effecting a reduction in fluorescence of ≥90% relative to the mean of replicate bacterium-only controls. MICs against nonreplicating *M. tuberculosis* were determined using a low-oxygen-recovery assay (LORA).

## 4. Conclusions

In summary, a series of novel 8-OMe CPFX-containing hybrids were designed, synthesized, and evaluated for their in vitro antimycobacterial and antibacterial activity. The results show that all of the 8-OMe CPFX-hydrozone hybrids (except for **7**) have potent activity against MTB H_37_Rv (MIC: <0.5 μM) which is better than the parent drug CPFX (MIC: 1.30 μM), some of the tested Gram-positive strains (MIC: 0.06–4 μg/mL), and most of the Gram-negative strains (MIC: ≤0.03–4 μg/mL). However, our results suggest that lipophilicity seems not to be an important parameter affecting both the anti-MTB and antibacterial activity.

## Figures and Tables

**Table 1:**
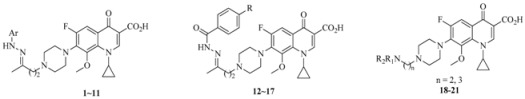
Structures and activity of compounds **1**–**21** against MTB H_37_Rv.

Compound.	Ar, R or R_1_R_2_N (n)	Clog *P* ^a^	MIC (μM)
**1**	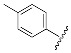	2.45	0.398
**2**	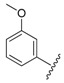	2.04	0.434
**3**	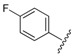	2.26	0.215
**4**	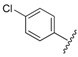	2.82	0.433
**5**	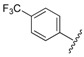	3.12	0.394
**6**	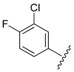	3.03	0.396
**7**	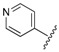	0.99	1.78
**8**	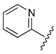	0.99	0.372
**9**	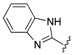	1.91	0.434
**10**	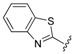	2.47	0.313
**11**	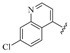	3.15	0.266
**12**	-H	0.99	0.226
**13**	-OCH_3_	1.27	0.214
**14**	-OH	0.66	0.210
**15**	-F	1.34	0.266
**16**	-Cl	1.90	0.272
**17**	-NO_2_	1.16	0.294
**18**	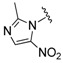 (2)	−0.13	1.72
**19**	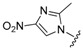 (3)	−0.01	60.3
**20**	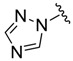 (2)	−1.16	4.10
**21**	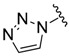 (2)	0.88	14.2
**CPFX**		−0.72	1.30
**MXFX**		−0.08	0.289
**INH**			0.336

^a^ The Clog P is calculated with ChemOffice 2012 software (CambridgeSoft, Cambridge, UK). CPFX, ciprofloxacin; MXFX, moxifloxacin; INH, isoniazid; MIC, minimum inhibitory concentration.

**Table 2:** In vitro antibacterial activity of compounds **1**–**21** against Gram-positive strains.

Compound	MIC (μg/mL)							
	MSSE	MRSE	MSSA	MRSA	E.fa.1	E.fa.2	E.fm.1	E.fm.2
**1**	0.25	64	0.25	0.25	1	1	64	64
**2**	0.25	>64	0.125	0.125	2	2	>64	>64
**3**	0.125	32	0.125	0.125	1	1	32	32
**4**	0.125	32	0.06	0.06	1	1	64	64
**5**	0.25	32	0.125	0.125	1	1	64	64
**6**	0.125	32	0.125	0.125	0.5	0.5	64	16
**7**	2	>64	2	2	8	8	>64	>64
**8**	0.125	32	0.125	0.125	1	1	64	64
**9**	1	64	1	1	2	2	128	128
**10**	0.5	64	0.25	0.5	1	1	64	64
**11**	1	128	0.5	0.5	4	4	>128	>128
**12**	0.125	64	0.125	0.125	0.5	0.5	64	64
**13**	0.125	64	0.125	0.25	1	1	64	64
**14**	0.06	64	0.125	0.06	0.5	0.5	64	64
**15**	0.125	64	0.125	0.125	0.5	0.5	>64	>64
**16**	0.125	32	0.125	0.125	1	1	32	32
**17**	0.125	128	0.25	0.25	1	1	128	128
**18**	1	>128	1	1	2	2	>128	>128
**19**	32	>64	32	32	>64	>64	>64	>64
**20**	4	>128	4	4	16	16	>128	>128
**21**	2	>128	4	4	16	16	>128	>128
**Conjugate-1**	0.06 ^a^	0.06 ^a^	0.125 ^a^	0.25 ^a^	ND	ND	0.5 ^a^	0.5 ^a^
**CPFX**	0.125	64	0.25	0.25	0.5	0.5	>128	>128
**LVFX**	0.125	32	0.125	0.125	1	0.5	32	32

Abbreviations: MSSE, methicillin-sensitive *Staphylococcus epidermidis* ATCC 12228; MRSE, methicillin-resistant *Staphylococcus epidermidis* 13-3; MSSA, methicillin-sensitive *Staphylococcus aureus* ATCC 29213; MRSA, methicillin-resistant *Staphylococcus aureus* ATCC 33591; E.fa.1, *Enterococcus faecalis* ATCC 29212; E.fa.2, *Enterococcus faecalis* ATCC 51299; E.fm.1, *Enterococcus faecium* ATCC 700221; E.fm.2, *Enterococcus faecium* 13-7; CPFX, ciprofloxacin; LVFX, levofloxacin; ^a^ the data was available from reference [24]; ND, not determined.

**Table 3:** In vitro antibacterial activity of compounds **1**–**21** against Gram-negative strains.

Compound	MIC (μg/mL)														
	E.co.1	E.co.2	K.p.1	K.p.2	P.a.	A.c.	E.c.	E.a.	S.m.1	M.m.	P.r.	P.v.	P.m.	S.m.2	C.f.
**1**	0.06	64	2	0.25	2	1	0.06	0.5	0.5	≤0.03	0.06	0.06	0.25	4	0.25
**2**	0.125	>64	8	2	8	2	0.25	0.5	2	0.125	0.06	0.06	0.5	16	0.25
**3**	0.06	32	4	0.25	1	1	0.06	0.25	0.5	0.06	0.06	0.06	0.25	8	0.125
**4**	0.06	32	4	0.25	4	1	0.125	0.25	0.5	0.06	0.06	0.06	0.5	4	1.125
**5**	0.125	>64	8	0.5	4	1	0.125	0.25	1	0.125	0.125	0.125	0.5	8	0.25
**6**	0.06	32	4	0.25	4	0.5	0.06	0.25	1	≤0.03	≤0.03	0.06	0.25	4	0.125
**7**	1	>64	64	4	32	16	2	8	8	1	1	1	8	32	2
**8**	≤0.03	32	4	0.25	2	0.25	0.06	0.25	0.25	≤0.03	≤0.03	≤0.03	0.25	2	0.06
**9**	0.5	>128	16	1	16	4	0.5	1	2	0.5	0.25	0.25	2	16	1
**10**	0.125	128	4	0.5	4	2	0.25	0.5	1	0.06	0.06	0.06	0.125	16	0.125
**11**	0.5	>128	64	4	16	8	0.5	2	4	0.5	0.25	0.5	1	32	1
**12**	≤0.03	32	2	0.06	2	0.5	0.06	0.125	0.25	0.06	≤0.03	≤0.03	0.125	2	0.06
**13**	0.06	16	2	0.25	8	0.5	0.06	0.25	0.5	≤0.03	≤0.03	≤0.03	0.25	4	0.125
**14**	≤0.03	32	2	0.125	1	0.5	0.06	0.25	0.25	≤0.03	≤0.03	≤0.03	0.25	2	0.06
**15**	0.06	64	2	0.25	2	0.5	≤0.03	0.25	0.25	≤0.03	0.06	≤0.03	0.25	2	0.06
**16**	≤0.03	32	4	0.25	2	0.5	0.06	0.125	0.5	≤0.03	≤0.03	0.06	0.5	0.5	0.125
**17**	0.06	64	2	0.125	4	0.5	≤0.03	0.25	0.25	0.06	≤0.03	≤0.03	0.25	4	0.06
**18**	0.125	128	8	2	8	2	0.125	0.5	1	0.125	0.06	0.06	1	16	0.25
**19**	8	>64	>64	32	>64	>64	8	32	64	8	4	4	32	>64	16
**20**	0.5	>128	32	4	64	16	1	4	8	1	0.5	0.5	4	64	2
**21**	1	>128	64	2	64	16	1	2	8	0.5	0.5	0.5	2	64	1
**Conjugate-1**	0.25 ^a^	1 ^a^	0.5 ^a^	1 ^a^	0.5 ^a^	ND	0.25 ^a^	ND	ND	ND	ND	ND	ND	ND	ND
**CPFX**	≤0.03	16	0.5	≤0.03	0.25	0.5	≤0.03	≤0.03	0.06	≤0.03	≤0.03	≤0.03	≤0.03	4	≤0.03
**LVFX**	≤0.03	16	0.5	≤0.03	1	0.125	≤0.03	0.06	0.125	≤0.03	≤0.03	≤0.03	0.06	1	≤0.03

Abbreviations: E.co.1, *Escherichia coli* ATCC 25922 ESBLs(-); E.co.2, *Escherichia coli* 14-11 ESBLs(+); K.p.1, *Klebsiella pneumoniae* ATCC 700603 ESBLs(+); K.p.2, *Klebsiella pneumoniae* 7 ESBLs(-); P.a., *Pseudomonas aeruginosa* ATCC 27853; A.c., *Acinetobacter calcoacetious* ATCC 19606; E.c., *Enterobacter cloacae* ATCC 43560; E.a., *Enterobacter aerogenes* ATCC 13048; S.m.1, *Serratia marcescens* ATCC 21074; M.m., *Morganella morganii* ATCC 25830; P.r., *Providentia rettgeri* ATCC 31052; P.v., *Proteus vulgaris* ATCC 29905; P.m., *Proteus mirabilis* 13-1; S.m.2, *Stenotrophomonas maltophilia* ATCC 13636; C.f., *Citrobacter freundii* ATCC 43864. ESBLs(+): Extended spectrum beta-lactamases (ESBLs)-producing. CPFX, ciprofloxacin; LVFX, levofloxacin; ^a^ the data was available from reference [24]; ND, not determined.
